# Impact of a telemedicine center on reducing the carbon footprint for primary health care: a multicenter retrospective cohort study

**DOI:** 10.1590/1516-3180.2025.3299.01102025

**Published:** 2025-12-15

**Authors:** Mônica Rossatti Molina, Marcus Vinicius Dutra Zuanazzi, Francisco Antônio Sousa de Araújo, Cleyton Zanardo de Oliveira, Vivian Oliveira Balan, Rafael Saad Fernandez, Camilla do Rosario Nicolino Chiorino, Eduardo Augusto Oliveira Barrozo, Dante Dianezi Gambardella, Soraya Camargo Ito Süffert

**Affiliations:** I Physician; Medical Coordinator, PROADI-SUS, BP – A Beneficência Portuguesa de São Paulo, São Paulo (SP), Brazil.; II Physician; Medical Consultant, TeleNordeste, PROADI-SUS, BP – A Beneficência Portuguesa de São Paulo, São Paulo (SP), Brazil.; III Economist; Econometrician, BP – A Beneficência Portuguesa de São Paulo, São Paulo (SP), Brazil.; IV Data and Statistics Lead, BP – A Beneficência Portuguesa de São Paulo, São Paulo (SP), Brazil.; V Senior Data Analyst, PROADI-SUS, BP – A Beneficência Portuguesa de São Paulo, São Paulo (SP), Brazil.; VI Project Coordinator, PROADI-SUS, BP – A Beneficência Portuguesa de São Paulo, São Paulo (SP), Brazil.; VII Nurse; Project Manager, BP – A Beneficência Portuguesa de São Paulo, São Paulo (SP), Brazil.; VIII Project Consultant, Government Relations, Social Responsibility and Sustainability, BP – A Beneficência Portuguesa de São Paulo, São Paulo (SP), Brazil.; IX Economist, BP – A Beneficência Portuguesa de São Paulo, São Paulo (SP), Brazil.; X Physician; Medical Researcher, TeleNordeste, PROADI-SUS, BP – A Beneficência Portuguesa de São Paulo, São Paulo (SP), Brazil.

**Keywords:** Carbon footprint, Telemedicine, Environmental health, Greenhouse gases, Public health, Environmental impact, Sustainability

## Abstract

**BACKGROUND:**

Telemedicine can promote access to specialized care and avoid travel to referral centers.

**OBJECTIVES:**

To present the environmental impacts and the positive results for the sustainability of the Brazilian public health system after the implementation of the TeleNordeste Project developed by hospital BP – A Beneficência Portuguesa de São Paulo.

**DESIGN AND SETTING:**

A retrospective cohort study was developed in three states in the Brazilian Northeast, Alagoas, Maranhão, and Piauí.

**METHODS:**

This study was conducted between August 2022 and December 2023. All patients participating in telemedicine care were selected for this type of care by Primary Health Care (PHC) doctors according to the need for clinical discussion. The variables analyzed were the total distance and time (round trip) saved by telemedicine care, the amount of carbon emissions not released into the environment, gasoline costs, resolution of care through teleconsultation, and evaluation of the Net Promoter Score.

**RESULTS:**

In total, 25,194 consultations were conducted via telemedicine, requiring in-person referral in 775 tele-interconsultations, representing a resolution rate of 96.92%. It saved approximately 10,737,287 miles (17,279,988.6 km) and 264,302 hours for patients and the municipal health department, and reduced carbon dioxide (CO_2_) emissions according to Environmental Protection Agency (EPA) parameters, estimated at 4,294,915 kg, saving US$ 1,660,068.89 (R$ 8,532,754.09) on gasoline.

**CONCLUSION:**

To our knowledge, in Brazil, this study is one of the first to present results on the impact of telemedicine on reducing carbon emissions in relation to the movement of patients to reference centers in healthcare networks and the resolution of care provided in health units in the context of the PROADI-SUS TeleNordeste Project developed by BP and promotes reflection on the potential benefits of telemedicine according to current evidence.

## INTRODUCTION

 Globally, health services are the centers of operations for healthcare and assistance, saving lives and promoting well-being; however, the current structure of these services involves the production of high amounts of carbon dioxide (CO_2_) through the use of significant resources and equipment that consume a lot of energy and harm the environment and public health.^1 -4^ Recent evidence estimates that healthcare systems represent approximately 4.4% of CO_2_ emissions worldwide, with higher percentages in industrialized countries.^3-7^ Recognition of this issue is beginning to gain prominence in the global scientific community through the science of sustainability in healthcare, which analyzes various dimensions of resource consumption and environmental emissions associated with healthcare activities. This emerging field of knowledge presents tools and metrics to quantify the unintended consequences of healthcare related to service provision, evaluate effective alternatives that improve patient care and safety, and simultaneously consider the sustainability of health systems.^2^

 Considering the ethical principle of non-maleficence,^8^ health professionals are committed to not causing harm,^6^ and in this way, health systems should lead the way towards greater sustainability and preservation of the environment and well-being of the population with proposals for alternatives for reducing CO_2_ emissions and maintaining quality of care.^1^ Telemedicine saw a rapid expansion of its use with the COVID-19 pandemic, enabling care to be maintained with less patient exposure.^9^ Since this period, it has had a positive impact on global healthcare, promoting greater access for many patients, especially for residents of distant areas of health centers, by reducing travel.^3^

 In this context, the hospital BP – A Beneficência Portuguesa de São Paulo, recognized as a hospital of excellence by the Brazilian Ministry of Health, through the Programa de Apoio ao Desenvolvimento Institucional do Sistema Único de Saúde (PROADI-SUS) has developed TeleNordeste Project, providing specialized consultations to several municipalities, in the Brazilian Northeast, to promote access to specialized care for users of the SUS and to avoid travel to reference centers with significant distances on a continental country like Brazil. Therefore, meeting the purpose of improving access to specialized assistance is associated with reducing CO_2_ emissions and their environmental impacts.^10^

 This study aimed to present the environmental impacts and the positive results for the sustainability of the public health system, SUS, benefiting users and managers of the locations served after the implementation of the TeleNordeste Project developed by the hospital BP – A Beneficência Portuguesa de São Paulo. 

## METHODS

### Study design

 A retrospective cohort study was conducted to analyze the environmental impacts of teleconsultations conducted through BP’s TeleNordeste Project. This study followed the guidelines of the "Strengthening the Reporting of Observational Studies in Epidemiology" (STROBE) for cohort studies.^11^

### Location and period of study

 This research was developed by the hospital BP – A Beneficência Portuguesa de São Paulo through the PROADI-SUS in partnership with the Ministry of Health, with the implementation of the Specialized Medical Assistance Project in the Northeast region of Brazil by means of Telemedicine, TeleNordeste from BP, registered with NUP 25000.170151/2021-65. 

 The project was implemented in 360 municipalities in three states in the Brazilian Northeast: Alagoas (90), Maranhão (134), and Piauí (136), with the proposal to carry out tele-interconsultations from August 2022 to December 2023. Synchronous tele-interconsultations were offered with specialist doctors, connecting doctors from different BP specialties, Primary Health Care (PHC) teams, and patients from the territories served in the same virtual environment synchronously, enabling discussions about clinical conditions dedicated to the management of the patient’s longitudinal care.^10^ Schedules of tele-interconsultations were carried out through the Bookings platform by PHC, and virtual meetings were carried out through the Teams platform, both from BP’s institutional Microsoft 365^®^ (Redmond, Washington State). 

 All tele-interconsultations conducted between August 2022 and December 2023 were included in the study. All patients participating in telemedicine care were selected for this type of care by PHC doctors according to the need for clinical discussion. 

 The specialist doctors hired by BP also carried out their work remotely from the home office, with a strategic alignment to contribute to reducing the project’s carbon emissions, although the impact of the specialized team was not analyzed in this study. 

 The resolution rate was calculated by dividing the number of tele-interconsultations that resulted in a complete specialized care visit with guidance on follow-up within the primary health care system by the total number of tele-interconsultations conducted by the project during the study period. The evaluation of service satisfaction in the TeleNordeste project was conducted through the application of the Net Promoter Score (NPS), a methodology used to evaluate customer satisfaction in relation to the service provided.^12,13^ The responses were categorized into three groups: promoters, neutrals, and detractors. The structured question was, "How likely are you to recommend TeleNoredeste to a friend?" Each participating PHC doctor received the question by email, and each patient received the question via a message on WhatsApp. 

### Variables

 The variables analyzed by the project were the total distance and total time (round trip) saved by telemedicine care, the amount of carbon emissions not released into the environment, the cost of gasoline, care resoluteness through tele-interconsultation, which indicates the completion of care without the need for referral to a specialized in-person consultation, and evaluation of the NPS. 

### Data analysis

 According to documents provided by the State Health Departments of Alagoas, Maranhão, and Piauí, references for specialized care were identified for referrals from Basic Health Units, making it possible to establish estimates of travel required to carry out in-person consultations with specialist doctors. Referred patients are usually transported to a reference point in the capital of each state in cars or minivans, as defined by the municipal departments of each city. It was assumed that all treated patients waited for a specialized face-to-face consultation and would leave the central point of the municipality with a round-trip by car to the reference capital. All patients included in the study were registered with the PHC. 

 The distance traveled in kilometers and times (in minutes) were calculated in May 2024 using data available on Google^®^ (Menlo Park, California). Briefly, the locations were identified by latitude and longitude, and the distance between two locations was calculated by establishing an available route involving highways with the shortest distance and travel time. It is important to note that the locations served by the project also had ferries along their route. Of the 360 municipalities participating in the project, sixteen municipalities (4.44%) required a ferry to access specialized references. 

 Traveling patients to specialized care involves several variables, such as the scheduling date and address of the reference outpatient clinic for each specialty, and companions are often necessary for children, older adults, and patients with special needs. Therefore, it was difficult for a car to transport these four patients to the references. In this way, the estimate of this study considered travel for each tele-interconsultation individually, but considering the optimization of the variables presented, the costs were also estimated if the car was fully occupied. This study calculated the distance traveled in a scenario of four passengers for a UBS that conducted four or more tele-interconsultations. For appointments of < 4, occupancy was adjusted according to the number of teleconsultations. 

 The CO_2_ emissions saved from vehicle travel were calculated using the Environmental Protection Agency (EPA) emissions parameters, which estimate that the average passenger vehicle emits about 400 grams of CO_2_ per mile, and the CO_2_ emissions from a gallon of gasoline are 8,887 grams of CO _2_/gallon.^14^ The United States Regular Gasoline Price^15^ data was used as of June 17, 2024, with the price of gasoline being US$ 3.435 per gallon, to calculate the estimated gasoline costs saved. 

 To convert into reality, this study used data from the Ministry of Finance with dollar value for conversion to the real for June 2024, worth US$1, quoted as R$ 5.14. 

 Statistical analysis was performed using the PSPP-GNU^®^ statistical software, GNU General Public License, version June 3 29, 2007. Continuous variables without a normal distribution were presented as medians and interquartile ranges. 

### Approval by the research ethics committee

 The study protocol was approved by the Ethics and Research Committee of the hospital BP – A Beneficência Portuguesa de São Paulo, and approved under number CAAE 72813923.6.0000.5483, with waiver of informed consent. 

## RESULTS

**Figure 1:**
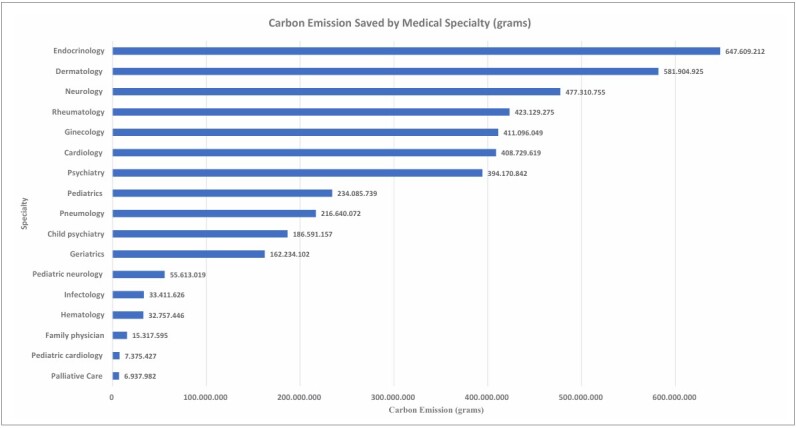
Carbon emission saved by medical specialty (grams).

 From August 2022 to December 2023, 25,194 teleinterconsultations were conducted via telemedicine, requiring in-person referral in 775 tele-interconsultations, representing a resolution of 96.92% of the clinical conditions treated by BP specialists in partnership with PHC doctors. Travel from PHC units to reference services was avoided, saving approximately 10,737,287.12 miles (equivalent to 17,279,988.6 km) for patients and municipal health departments, with an estimated time savings of 264,302 h. Regarding the reduction of CO_2_ emissions according to EPA parameters, it was approximately 4,294,915 kg of CO_2_ (**Tables 1** **and** **2**) and equivalent, according to the parameters, 483,280.61 gallons of gasoline, saving US$ 1,660,068.89 (R$ 8,532,754.09) on gasoline. The median distance saved was approximately 442 miles with an interquartile range of 308 miles (equivalent to 712 km and an interquartile range of 496). Regarding carbon emissions, the median amount of CO _2_ avoided was approximately 177 kg of CO_2_ , with an interquartile range of 123.28. If it were possible to optimize trips to reach a maximum occupancy of passengers per vehicle, the estimated distance would be 3,843,362.57 miles (equivalent to 6,185,292.5 km), representing approximately 172,988.07 gallons of gasoline. This would save US$ 594,214.03 (R$ 3,054,260.11). The reduction in CO_2_ emissions according to EPA parameters was approximately 1,537,345 kg of CO_2_ . The median distance saved was approximately 123 miles, with an interquartile range of 91.65 (equivalent to 198 km, with an interquartile range of 147.5). Regarding carbon emissions, the median CO_2_ avoided was approximately 49.21 kg of CO_2_ , with an interquartile range of 36.66 kg (**Table 1**). Carbon emission reductions by specialty are shown in **Figure 1**. 

**Table 1:** Variables according to the state

**State**	Number of teleconsultations*	In-person referral*	**Economy**			Resolution of teleconsultation**	Referral fee**
			Distance (miles)*	Time (hours)*	Carbon emission (grams)*	**Primary health care**	**Specialist consultation**
Alagoas	5,842	166	1,006,539.06	26,025.32	402,615,624	97.16%	2.84%
Maranhão	10,851	359	5,615,626.62	146,094.57	2,246,250,648	96.69%	3.31%
Piauí	8,501	250	4,115,121.44	92,182.20	1,646,048,576	97.06%	2.94%
Total	25,194	775	10,737,287.12	264,302.09	4,294,914,848	96.92%	3.08%

* Absolute values and

** Percentage (%)

**Table 2:** Variables according to specialties

**Specialty**	Number of teleconsultations*	In-person referral*	**Economy**			Resolution of teleconsultation**	Referral fee**
			Distance (miles)*	Time (hours)*	Carbon emission (grams)*	**Primary health care**	Specialist consultation **
Cardiology (AL)	661	11	103,760.54	2,708.33	41,504,215.38	98.34%	1.66%
Cardiology (MA)	1,031	20	539,105.62	13,985.53	215,642,249.30	98.06%	1.94%
Cardiology (PI)	807	5	378,957.89	8,562.87	151,583,154.40	99.38%	0.62%
**Cardiology total**	**2,499**	**36**	**1,021,824.05**	**2,556.73**	**408,729,619.08**	**98.56%**	**1.44%**
Pediatric cardiology (AL)	5	0	929.32	23.3	371,729.10	100%	0%
Pediatric cardiology (MA)	15	0	9,846.25	249.47	3,938,499.17	100%	0%
Pediatric cardiology (PI)	18	0	7,663.0	175.3	3,065,199.24	100%	0%
**Pediatric cardiology total**	**38**	**0**	**18,438.57**	**448.07**	**7,375,427.51**	**100%**	**0%**
Palliative care (AL)	11	0	1,594.44	45.06	637,775.40	100%	0%
Palliative care (MA)	5	0	3,084.49	78.5	1,233,794.64	100%	0%
Palliative care (PI)	23	0	12,666.03	278.77	5,066,412.15	100%	0%
**Palliative Care total**	**39**	**0**	**17,344.96**	**402.33**	**6,937,982.19**	**100%**	**0%**
Dermatology (AL)	887	61	154,619.40	4,009.77	61,847,759.09	93.12%	6.88%
Dermatology (MA)	1,553	142	811,642.01	21,167.53	324,656,804.30	90.86%	9.14%
Dermatology (PI)	1,030	67	488,505.09	10,972.90	195,400,361.90	93.5%	6.5%
**Dermatology total**	**3,470**	**270**	**1,454,762.31**	**36,150.20**	**581,904,925.29**	**92.22%**	**7.78%**
Endocrinology (AL)	1,033	7	188,427.21	4,886.33	75,370,884.04	99.32%	0.68%
Endocrinology (MA)	1,652	18	844,135.87	21,847.50	337,654,348.60	98.91%	1.09%
Endocrinology (PI)	1,246	10	586,459.95	13,142.66	234,583,979.60	99.2%	0.8%
**Endocrinology total**	**3,931**	**35**	**1,619,023.03**	**39,876.49**	**647,609,212.24**	**99.11%**	**0.89%**
Geriatrics (AL)	188	6	34,863.90	882.33	13,945,557.94	96.81%	3.19%
Geriatrics (MA)	510	9	266,473.80	6,815.83	106,589,517.20	98.23%	1.77%
Geriatrics (PI)	242	1	104,247.57	2,345.37	41,699,027.68	99.58%	0.42%
**Geriatrics total**	**940**	**16**	**405,585.27**	**10,043.53**	**162,234,102.8**	**98.29%**	**1.71%**
Gynecology (AL)	516	7	84,084.32	2,265.11	33,633,729.02	98.64%	1.36%
Gynecology (MA)	1,013	23	522,563.36	13,987.73	209,025,342	97.73%	2.27%
Gynecology (PI)	805	30	421,092.44	9,418.56	168,436,978	96.27%	3.73%
**Gynecology total**	**2,334**	**60**	**1,027,740.12**	**25,671.40**	**411,096,049.02**	**97.43%**	**2.57%**
Hematology (AL)	48	5	5,465.58	155.2	2,186,232.40	89.58%	10.42%
Hematology (MA)	96	1	45,270.62	1,182.13	18,108,247.83	98.96%	1.04%
Hematology (PI)	64	4	31,157.42	706.86	12,462,966.28	93.75%	6.25%
**Hematology total**	**208**	**10**	**81,893.62**	**2,044.20**	**32,757,446.51**	**95.19%**	**4.81%**
Infectology (AL)	32	0	5,508.95	143.64	2,203,581.09	100%	0%
Infectology (MA)	126	4	64,372.81	1,667.8	25,749,125.11	96.83%	3.17%
Infectology (PI)	31	4	13,647.30	306.03	5,458,919.91	87.1%	12.9%
**Infectology total**	**189**	**8**	**83,529.06**	**2,117.47**	**33,411,626.11**	**95.77%**	**4.23%**
Family physician (AL)	17	3	2,086.69	58.63	834,675.50	82.35%	17.65%
Family physician (MA)	41	4	21,244.68	542.6	8,497,872.43	90.24%	9.76%
Family physician (PI)	33	1	14,962.62	350.3	5,985,047.32	96.97%	3.03%
**Family physician total**	**91**	**8**	**38,293.99**	**951.53**	**15,317,595.25**	**91.21%**	**8.79%**
Neurology (AL)	724	13	127,414.15	3,224.88	50,965,660.54	98.2%	1.8%
Neurology (MA)	1,162	41	577,664.07	15,019.10	231,065,626.70	96.47%	3.53%
Neurology (PI)	1,030	37	488,198.67	10,974.87	195,279,467.90	96.41%	3.59%
**Neurology total**	**2,916**	**91**	**1,193,276.89**	**29,218.85**	**477,310,755.14**	**96.88%**	**3.12%**
Pediatric neurology (AL)	37	3	10,614.51	252.67	4,245,804.50	91.89%	8.11%
Pediatric neurology (MA)	126	3	63,541.42	1,629.33	25,416,567.25	97.62%	2.38%
Pediatric neurology (PI)	130	8	64,876.62	1,461.93	25,950,648.21	93.85%	6.15%
**Pediatric neurology total**	**293**	**14**	**139,032.55**	**3,343.93**	**55,613,019.96**	**95.22%**	**4.78%**
Pediatrics (AL)	242	21	40,871.93	1,068.40	16,348,773.16	91.32%	8.68%
Pediatrics (MA)	626	35	311,241.97	8,151.70	124,496,788.80	94.41%	5.59%
Pediatrics (PI)	409	35	233,100.44	5,251.43	93,240,177.36	91.44%	8.56%
**Pediatrics total**	**1,277**	**91**	**585,214.34**	**14,471.53**	**234,085,739.32**	**92.87%**	**7.13%**
Pneumology (AL)	260	9	47,177.36	1,199.43	18,870,943.69	96.54%	3.46%
Pneumology (MA)	531	19	255,336.96	6,633.53	102,134,782.90	96.42%	3.58%
Pneumology (PI)	492	7	239,085.86	5,331.46	95,634,345.42	98.58%	1.42%
**Pneumology total**	**1,283**	**35**	**541,600.18**	**13,164.42**	**216,640,072.01**	**97.27%**	**2.73%**
Psychiatry (AL)	486	12	81,784.38	2,129.8	32,713,751.69	97.53%	2.47%
Psychiatry (MA)	777	15	428,386.60	11,051.73	171,354,638.90	98.07%	1.93%
Psychiatry (PI)	963	24	475,256.13	10,529.13	190,102,451.70	97.51%	2.49%
**Psychiatry total**	**2,226**	**51**	**985,427.11**	**23,710.66**	**394,170,742.29**	**97.71%**	**2.29%**
Child psychiatry (AL)	153	3	26,330.73	661.96	10,532,291.42	98.04%	1.96%
Child psychiatry (MA)	515	6	276,558.65	7,198.16	110,623, 459	98.83%	1.17%
Child psychiatry (PI)	330	3	163,588.52	3,634.63	65,435,407.22	99.09%	0.91%
**Child psychiatry total**	**998**	**12**	**466,477.90**	**11,494.76**	**186,591,157.64**	**98.8%**	**1.2%**
Rheumatology (AL)	542	5	91,005.65	2,310.45	36,402,260.80	99.08%	0.92%
Rheumatology (MA)	1,072	19	575,157.46	14,886.36	230,062,982.20	98.23%	1.77%
Rheumatology (PI)	848	14	391,660.08	8,739.10	156,664,032.10	98.35%	1.65%
**Rheumatology total**	**2,462**	**38**	**1,057,823.19**	**25,935.91**	**423,129,275.10**	**98.46%**	**1.54%**

AL, Alagoas; MA, Maranhão; PI, Piauí;

* Absolute values

** Percentage (%)

 For the state of Alagoas, the median distance saved was approximately 139.2 miles with an interquartile range of 144.6 miles (equivalent to 224 km with an interquartile range of 232.7 km). Regarding carbon emissions, the median CO_2_ avoided was approximately 55.67 kg of CO_2_, with an interquartile range of 57.83. 

 For the state of Maranhão, the median distance saved was approximately 484.67 miles with an interquartile range of 221.2 (equivalent to 780 km with an interquartile range of 356 km). Regarding carbon emissions, the median amount of CO _2_ avoided was approximately 193.86 kg of CO_2_, with an interquartile range of 88.48. 

 For the state of Piauí, the median distance saved was approximately 492.13 miles with an interquartile range of 208.78 (equivalent to 792 km with an interquartile range of 336 km). Regarding carbon emissions, the median amount of CO _2_ avoided was approximately 196.85 kg of CO_2_ , with an interquartile range of 83.51 g. 

 The three specialties with the highest numbers of teleconsultations were Psychiatry, Endocrinology, and Dermatology. 

**Figure 2:**
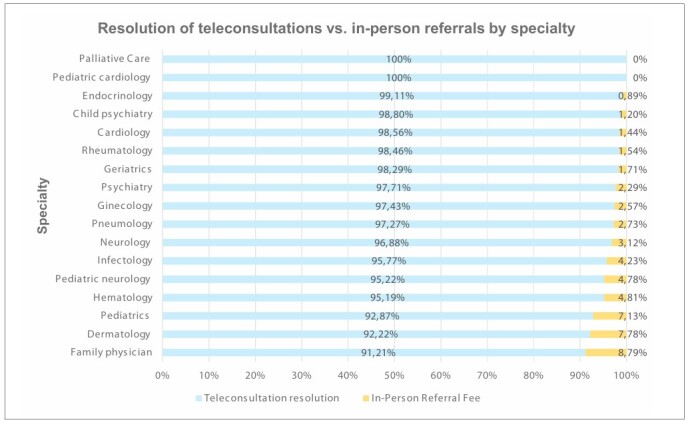
Resolution of teleconsultations versus in-person referrals by specialty.

 Some specialties, such as pediatric cardiology and palliative care, showed 100% resolution in clinical discussions. Additional information is presented in **Table 2** and **Figure 2**. 

 Regarding the evaluation of satisfaction with TeleNordeste services, the NPS of PHC medical professionals was 91, and the result related to patients served was 89.3, considering the three states served by the project. 

## DISCUSSION

 The implementation of the TeleNordeste project avoided the movement of patients to specialized references and promoted positive socioeconomic and environmental impacts, as there was a reduction in costs for municipal health departments, saving between US$ 594,214.03 (R$ 3,054,260.11) and US$ 1,660,068.89 (R$ 8,532,754.09) on gasoline, and a reduction in CO_2_ emissions for the environment between 1,537,345 and 4,294,915 kg, depending on transportation optimization. Besides the quantitative aspects, the project enabled qualitative gains for care provided through support for PHC, establishing greater resolution with overall results of 96.92% with agile and timely access, directly impacting the care of people using local healthcare networks, and developing a care repertoire of professionals benefiting from the intervention with positive NPS results for participating PHC users and doctors.^10^

 It is important to highlight that in-person referrals, established after evaluation by BP specialists, provided timely guidance for each tele-interconsultation and, when relevant, requests for examinations and therapeutic prescriptions were made, enabling the coordination of care until access to in-person consultation. In this context, this study highlights the experience of the project’s team of dermatologists, who referred 7.78% of patients evaluated in tele-interconsultation, and many of these clinical conditions required a biopsy to be carried out for an effective diagnostic conclusion; therefore, the indication for the procedure was guided by a specialist. 

 These results indicate a significant impact on carbon emissions, congruent with current evidence on the topic, which highlights that reducing face-to-face consultations has become a vital tool for sustainable health development.^5,16-18^ According to a systematic review carried out by Purohit, Smith, and Hibble^16^ in 2021, all studies analyzed unanimously reported that the use of telemedicine promoted a reduction in CO_2_ emissions from healthcare, mainly by reducing the costs associated with emissions during travel. Carbon footprint reduction ranged between 0.70 and 372 kg CO_2_ per consultation. 

 Furthermore, BP’s proposal to structure a remote work model for medical specialists contributed to the reduction of carbon emissions by the health service and is also a factor associated with less medical exhaustion, which can consequently improve the quality of patient care.^5^

 Considering the environmental impact related to the reduction of 4,294,915 kg of CO _2_ emissions promoted by the TeleNordeste project, according to the EPA calculator,^19^ these results are equivalent to carbon sequestration by 71,017 tree seedlings grown over 10 years, 5,014 acres of United States forests in one year, or 27,5 acres of United States Forests saved from conversion to farmland in one year. This is equivalent to the greenhouse gas emissions avoided by 1,491 tons of waste instead of landfilling or 186,554 trash bags of waste recycled instead of landfilling. 

 The advantages of telemedicine care reported by studies include not only carbon emissions but also lower financial costs, greater satisfaction, easier access for residents of rural areas, fewer appointments missed due to abstention,^16^ and reduced waiting times in referral queues.^20^ It is also important to highlight that, according to national medical demographics, Brazil reached 2.6 doctors per 1,000 inhabitants in 2023, but the distribution of doctors by region is diverse in the country, and the Northeast region has 1.93 doctors per 1,000 inhabitants. In 2022, 62.3% of doctors in Brazil were specialists.^21^ The project also presented itself as a possibility of access to specialized consultation. 

 The difficulty in accessing specialized consultation is a reality and mainly affects countries with distant reference centers for most populations. Constanzo et al.^20^ carried out a retrospective cohort study in Chile with 1743 patients seen through teleconsultation in neurology and found that the waiting time for care was < 60% for telemedicine than for in-person consultation. A descriptive study conducted in Recife by Aquino et al.,^22^ in 2022, identified an average waiting time in the regulation queue of 270 days, with a maximum of 750 days when patients were able to access teleconsultation in the neurology specialty offered during the COVID-19 pandemic. 

 The average resolution of teleconsultations was 96.92%, with some specialties presenting 100%, according to literature it is known that PHC is capable of resolving around 85% of clinical conditions,^23^ the possibility of discussion through telemedicine of the BP’s TeleNordeste Project increased resolution, delivered as result quality of life for those who waiting long periods for physicians specialists, and promoted a reduction in travel and, consequently, carbon emissions. 

 PHC integration with a telemedicine center is a strategy that has proven to be relevant in reducing the carbon footprint, even in a densely populated country with little use of automobiles, like Switzerland.^17^ This study presents the experience of a PROADI project in the SUS, enabling the evaluation of the impact of telemedicine on PHC in a continental country such as Brazil, which, besides the significant distances, the necessary means of transport involved, in the regions served by the project, required roads and/or ferries, making it difficult for many SUS users to travel to their specialized references. 

 Considering these aspects, an important concept is that prevention is the most effective means of ensuring the sustainability of health care from environmental, social, and economic aspects; health systems must devote attention to health promotion and disease prevention to the detriment of the focus of disease treatment,^2,24^ which requires policies that support PHC and public health, robust screening programs, fair and universal access to health resources, including financing models that align incentives bringing value to well-being and health.^2^ BP’s TeleNordeste project aims to promote timely and agile universal access to SUS users, articulating care pathways and assistance to improve health outcomes, aligned with the sustainability of health systems and environmental responsibility, presenting the first result of the socioeconomic and environmental impact of a telemedicine project implemented in the SUS. 

 This study had limitations. This study carried out an analysis of estimates of travel distances according to the shortest route, not presenting precise measurements of travel that would be carried out; however, many of the assumptions are conservative and, if wrong, would underestimate, rather than overestimate, reducing CO_2_ emissions. It was also not possible to estimate the amount of carbon emissions related to ferries used on routes from some municipalities to a specialized reference site. Furthermore, this study did not have access to the socioeconomic data of the patients treated; therefore, it was not possible to calculate individual patient savings related to travel and reduction in work absences, considering the indirect impacts of the project. 

## CONCLUSIONS

 To our knowledge, in Brazil, this study is one of the first to present results on the impact of telemedicine on the reduction of carbon emissions, savings for Municipal Health Departments regarding patient travel to reference centers in Health Care Networks and the resolution of care provided in the context of the PROADI-SUS TeleNordeste Project developed by hospital BP – A Beneficência Portuguesa de São Paulo, and promotes reflection on the potential benefits of telemedicine as a valuable opportunity to promote the sustainability of the Health System in a continental country and the need for continued investment in digital health technologies such as strategy to promote health and preserve the environment. 

## Data Availability

All data generated or analyzed during this study are included in this published article.
